# Amyloidophilic Molecule Interactions on the Surface of Insulin Fibrils: Cooperative Binding and Fluorescence Quenching

**DOI:** 10.1038/s41598-019-56788-y

**Published:** 2019-12-30

**Authors:** Mantas Ziaunys, Kamile Mikalauskaite, Vytautas Smirnovas

**Affiliations:** Institute of Biotechnology, Life Sciences Center, Vilnius University, Vilnius, Lithuania

**Keywords:** Protein aggregation, Fluorescence spectroscopy, Spectrophotometry

## Abstract

Protein aggregation into insoluble fibrillar aggregates is linked to several neurodegenerative disorders, such as Alzheimer’s or Parkinson’s disease. Commonly used methods to study aggregation inhibition or fibril destabilization by potential drugs include spectroscopic measurements of amyloidophilic dye molecule fluorescence or absorbance changes. In this work we show the cross-interactions of five different dye molecules on the surface of insulin amyloid fibrils, resulting in cooperative binding and fluorescence quenching.

## Introduction

The aggregation of amyloidogenic proteins into highly structured amyloid fibrils is linked to several neurodegenerative disorders, such as Alzheimer’s, Parkinson’s and prion diseases^1–3^. The process of amyloid formation consists of native protein structural rearrangement and subsequent elongation into insoluble fibrillar aggregates^4^. Besides being cytotoxic^5^, these fibrils possess the ability to self-replicate, a process which is being extensively studied in the hopes of understanding its mechanism and finding potential inhibitors^6,7^. However, as of yet, there is still no known cure or effective treatment available^8^.

*In vitro* experiments of amyloid fibril formation and self-replication are often used when screening for potential inhibitory molecules or compounds that cause a disassembly of the aggregate structure. These observations are typically done by employing one or several methods, such as atomic force microscopy^9^, extrinsic dye fluorescence spectroscopy^10^, light scattering^11^, Fourier-transform infrared spectroscopy^12^, circular dichroism spectroscopy^13^ and others^14^. While all these methods have their advantages and shortcomings, in this work we will focus on the use of absorbance and fluorescence spectroscopy. There is a growing amount of research being conducted on amyloid fibril aggregation and dissociation with the use of several amyloidophilic dye molecules. The most frequently utilized ones are thioflavin-T (ThT) and Congo red (CR) – which bind to grooves formed by beta-sheets on the fibril’s surface^15–18^, as well as 8-anilinonaphthalene-1-sulfonic acid (ANS) – which binds to the fibril’s hydrophobic regions^19^. A common assumption made when using these dyes is that a change in their fluorescence signal or shift in their absorbance spectrum is the result of either amyloid fibril formation or dissociation^20–23^.

A few recent reports allude to the fact that the use of such extrinsic fibril dyes is problematic when screening for potential aggregation inhibiting or fibril destabilizing substances. In the work done by Girych *et al*.^24^ it is shown that when two of these amyloidophilic dyes are used in unison, namely ThT and CR, the fluorescence intensity of ThT is greatly reduced. CR is also known to quench other compound fluorescence, as shown by Patel *et al*.^25^. A report by Buell *et al*.^26^ demonstrates that ThT and CR interact with one another at neutral pH and influence each other’s binding to fibrils. The work by Hudson *et al*.^27^ displays a ThT fluorescence quenching effect by polyphenolic anti-amyloid compounds. Another report by Lindberg *et al*.^28^ has shown that when an increasing number of ThT molecules bind to the surface of fibrils, they cause a self-quenching effect. This indicates that the presence of another molecule, capable of binding to the fibril’s surface, may alter the dye’s fluorescence potential, thus leading to false conclusions that the compound’s addition resulted in the inhibition or disassembly of amyloid fibrils.

Potential drugs that directly influence amyloid formation or dissociation have to, in one way or another, interact with either the native state protein (stabilizing the native state or changing the aggregation’s path to non-fibrillar species^29,30)^ or with the fibril (causing its dissociation or preventing it from incorporating native proteins^31,32)^. In cases when it interacts with fibrils, it could act in a similar way as CR did with ThT in the previously mentioned reports. This could lead to a false identification of the molecule in question as a potential anti-amyloid drug, while in actuality, its only function was as a fluorescence quencher.

In this work we examine five amyloidophilic dye molecules possessing very characteristic spectral properties – thioflavin-T, Congo red, 8-anilinonaphthalene-1-sulfonic acid, dapoxyl (Dap)^33^ and methylene blue (MB)^34,35^ in order to determine their cross-interactions when binding to insulin amyloid fibrils. We show the changes of absorbance spectra when these molecules interact with one another in solution and on fibrils, as well as how they influence each other’s fibril binding affinity and the fluorescence intensity of ThT, ANS and Dap.

## Methods

### Fibril preparation

Human recombinant insulin (Sigma-Aldrich cat. No. 91077 C) was dissolved in PBS (pH 7.4) buffer to a final protein concentration of 300 µM (1.74 mg/ml). Insulin concentration was determined by measuring the sample’s absorbance at 280 nm, with ε = 6335 M^−1^cm^−1^ and M = 5808 Da. The protein solutions were distributed to test tubes (Fisher cat. No. 15432545) at a volume of 1 mL each and 2 glass beads (Merck, cat. No. 104015) were placed inside every tube. The samples were then mixed at a constant 600 rpm agitation at 60 °C in a Ditabis ThermoMixer MHR 23 for 24 hours. Formation of amyloids was confirmed by both atomic force microscopy (AFM) (Supplementary Fig. S1), which showed short insulin fibrils and Fourier-transform infrared spectroscopy (FTIR) (Supplementary Fig. S1), which revealed a beta-sheet secondary structure^36^.

### Dye solution preparation

ThT, CR, ANS, Dap and MB powders were dissolved in PBS (pH 7.4) buffer, filtered and diluted to a final concentration of 150 µM for ThT, CR, ANS, Dap and 20 µM for MB. Dye concentrations were determined by measuring a 10-fold diluted sample’s absorbance at 412 nm for ThT (ε = 22.1 × 10^3^ M^−1^cm^−1^), 486 nm for CR (ε = 33.3 × 10^3^ M^−1^cm^−1^), 351 nm for ANS (ε = 5.2 × 10^3^ M^−1^cm^−1^), 348 nm for Dap (ε = 28.4 × 10^3^ M^−1^cm^−1^) and 664 nm for MB (ε = 73.1 × 10^3^ M^−1^cm^−1^). The stock solutions were kept at 4 °C in the dark to prevent oxidation. Dye absorbance spectra before and after the experiments was measured to determine if there was no oxidation or loss of spectral properties.

### Absorbance measurements

Samples for absorbance measurements were prepared by mixing dye, fibril and PBS solutions so that the dye and fibril solutions would be diluted 3 times, yielding 300 µL samples containing either a single dye, two dyes or dyes with 100 µM of insulin fibrils. The samples were then incubated at room temperature for 1 hour, after which they were scanned using a Shimadzu UV-1800 spectrophotometer (1 nm steps). The samples were diluted either 2 or 3 times when their absorbance was over the value of 2 in the scanned range. For each condition, three separate measurements were taken and a spectrum of insulin fibrils without dyes was subtracted. Then the samples were centrifuged at 20’000 g for 20 min. 150 µL of supernatant was carefully removed from each sample and its absorbance spectrum was scanned (1 nm steps). For each condition, three samples were scanned three times and the 9 spectra were averaged.

### Absorbance difference and compensation curves

The absorbance spectra of two combined dyes were subtracted from the combined spectra when dyes were measured separately. In order to determine the contribution of each respective dye molecule to the resulting difference, the following system of equations was used:

1 $${{\rm{\varepsilon }}}_{11}\times {\rm{x}}+{{\rm{\varepsilon }}}_{12}\times {\rm{y}}={{\rm{A}}}_{1}$$

2 $${{\rm{\varepsilon }}}_{21}\times {\rm{x}}+{{\rm{\varepsilon }}}_{22}\times {\rm{y}}={{\rm{A}}}_{2}$$

where ε_11_ is the first dye’s extinction coefficient at wavelength λ_1_, ε_12_ is the second dye’s extinction coefficient at wavelength λ_1_, ε_21_ is the first dye’s extinction coefficient at wavelength λ_2_, ε_22_ is the second dye’s extinction coefficient at wavelength λ_2_, A_1_ is the sample’s absorbance at wavelength λ_1_, A_2_ is the sample’s absorbance at wavelength λ_2_, x is the concentration of the first dye and y is the concentration of the second dye. The specific wavelengths were chosen based on each molecule’s specific absorbance maxima described in the dye solution preparation section, with an exception for the ANS and Dap pair, where the wavelength used for ANS was 265 nm (ε = 16.2 × 10^3^ M^−1^cm^−1^), due to the close proximity of both absorbance maxima (351 nm and 348 nm for ANS and Dap respectively).

The contribution of each dye to the absorbance difference was then used to calculate a compensation curve, by combining each dye’s respective absorbance fraction, i.e. if the difference was the result of a loss of 20% ThT and 10% ANS, then the compensation curve was the sum of 20% ThT and 10% ANS spectra. To correct for this difference in dye-fibril interaction experiments, the absorbance intensity at every wavelength is multiplied by the ratio between the absorbance intensities of dyes measured separately and together at the corresponding wavelength.

### Fluorescence measurements

For fluorescence measurements, samples were prepared as described in the absorbance measurement section, apart from the last centrifugation step. Excitation-emission matrices (EEM) of each sample were then scanned using a Varian Cary Eclipse fluorescence spectrophotometer, using an excitation and emission range from 200 to 800 nm, with 10 nm excitation and 2 nm emission steps using 5 nm excitation and 2.5 nm emission slit widths. From each EEM, 100 nm x 100 nm regions containing fluorescence signals were scanned in greater detail with 1 nm excitation and 2 nm emission steps, using the same slit widths. For every condition, three detailed EEMs were scanned and averaged. The signal resulting from Rayleigh scattering was subtracted from each EEM.

### Inner filter corrections

To correct for both the primary and secondary inner filter effects, the absorbance spectra of samples used in the fluorescence measurements were taken. In cases when the absorbance values were greater than 2, the samples were diluted either 2 or 3 times. Fibril absorbance spectra were then subtracted from the dye-fibril spectra to determine the absorbance of free and bound dye present in the solution. Each EEM was corrected for the inner filter effect by using the following equation:

3 $${{\rm{I}}}_{{\rm{m}}}={{\rm{I}}}_{{\rm{c}}}\times {10}^{-(({\rm{AEx}}+{\rm{AEm}})/2)}$$

where AEx is the sample’s absorbance at the excitation wavelength, AEm is the sample’s absorbance at the emission wavelength, I_m_ is the signal intensity observed during measurement and I_c_ is the corrected signal intensity.

### Titration of ThT-fibrils with CR

Insulin fibrils were combined with ThT and CR solutions to a final fibril concentration of 100 µM, ThT concentrations – 50, 100 and 150 µM and CR concentrations in the range from 0 to 50 µM. Samples were incubated for 1 hour, after which their fluorescence was measured with an excitation wavelength of 440 nm and 460–500 nm emission range using 5 nm excitation and 2.5 nm emission slits. Absorbance was measured after diluting a fraction of the solution 10 times. Non-bound ThT concentration was determined after sample centrifugation as described previously. Non-bound CR was determined to be less than 1 µM in all of the supernatants.

## Results

### Dye molecule interactions

**Figure 1:**
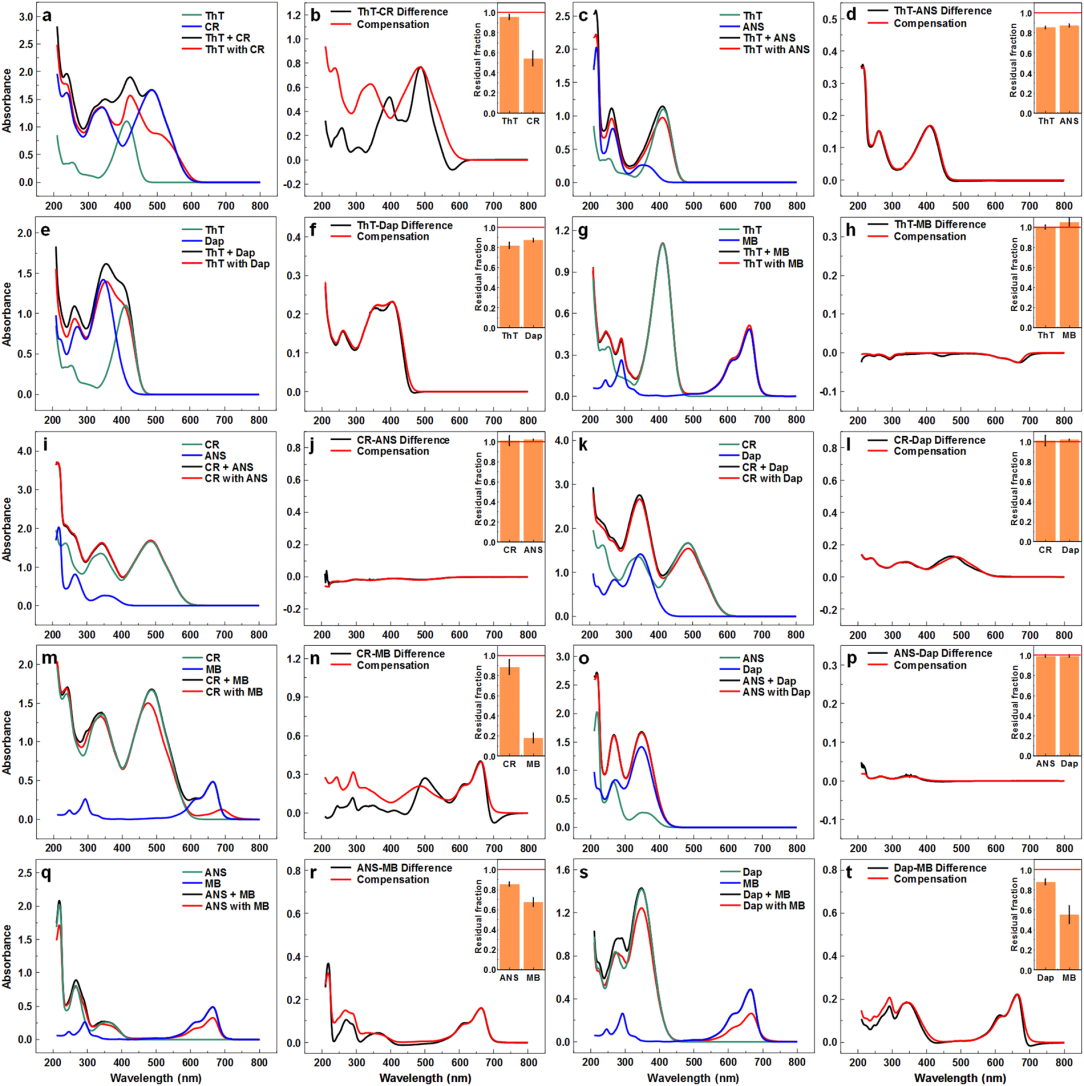
Absorbance spectra of ThT, CR, ANS, Dap and MB pairs. Comparison of absorbance spectra when dyes are measured separately and the spectra are combined (marked as Dye + Dye) and when they are measured together (marked as Dye with Dye), as well as the difference between both spectra and a calculated compensation curve for ThT and CR (**a**,**b**), ThT and ANS (**c**,**d**), ThT and Dap (**e**,**f**), ThT and MB (**g**,**h**), CR and ANS (**i**,**j**), CR and Dap (**k**,**l**), CR and MB (**m**,**n**), ANS and Dap (**o**,**p**), ANS and MB (**q**,**r**), Dap and MB (**s**,**t**). Inserts show the residual fraction of each dye’s respective absorbance remaining when the dyes are combined as opposed to being scanned separately.

Before any dye-fibril binding could be examined, the interactions between amyloidophilic molecules had to be evaluated. For each pair, a combined dye solution spectrum was measured and compared against a sum of corresponding separate dye solution spectra with identical concentrations. In some cases, we observed a massive disparity between the spectra, as seen with ThT-CR (Fig. 1a,b) and CR-MB (Fig. 1m,n). In other cases, there is a reduction in overall absorbance, as seen with ThT-ANS (Fig. 1c,d), ThT-Dap (Fig. 1e,f), ANS-MB (Fig. 1q,r) and Dap-MB (Fig. 1s,t). For other dye pairs, there is almost no difference between the spectra, as seen with ThT-MB (Fig. 1g,h), CR-ANS (Fig. 1I,j), CR-Dap (Fig. 1k,l) and ANS-Dap (Fig. 1o,p).

For each dye-pair absorbance spectra difference, a compensation spectrum was created by calculating what fraction of each dye is needed to compensate for the change in absorbance. In most cases, the difference and compensation spectra overlap, however, in the case of ThT-CR, CR-MB and to a lower extent ANS-MB and Dap-MB, there appears to be no way to compensate for this change. Examining how much of each dye is needed to create the compensation spectra, considerable fractions are required for ThT-CR, ThT-ANS, ThT-Dap, CR-MB, ANS-MB and Dap-MB pairs. In the case of ThT-MB, the combined dye solution creates the appearance that there is slightly more MB dye present than when measured separately. These interactions have to be taken into consideration in further dye-fibril binding experiments by correcting dye-pair spectra as described in the method section.

### Dye interaction with fibrils

**Figure 2:**
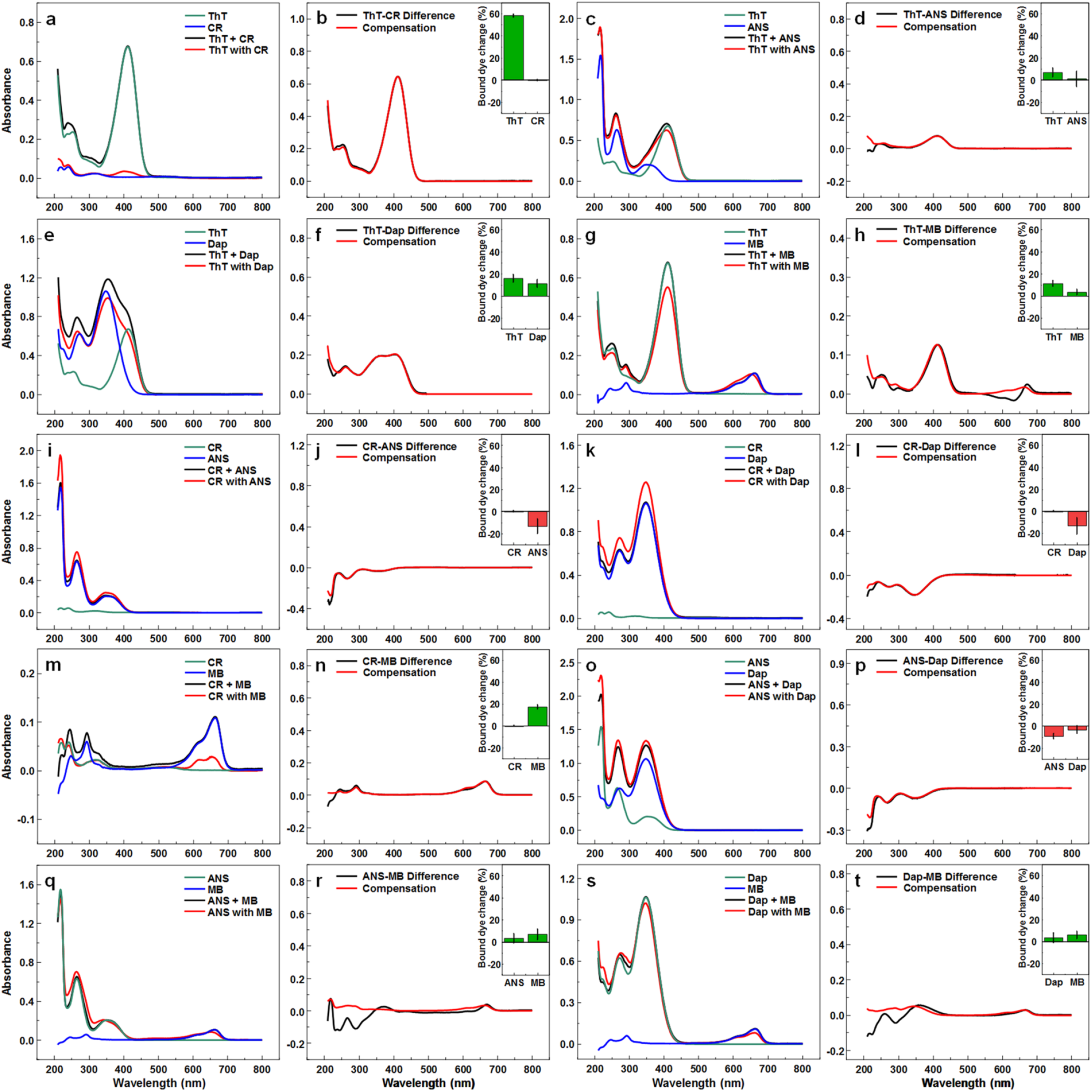
Absorbance spectra of ThT, CR, ANS, Dap and MB pairs remaining in supernatant after separation from fibrils. Comparison of dye supernatant absorbance spectra when the dyes were mixed with fibrils separately and the spectra were then combined (marked as Dye + Dye) and when both dyes were mixed with fibrils (marked as Dye with Dye), as well as the difference between both spectra and a calculated difference compensation curve for ThT and CR (**a**,**b**), ThT and ANS (**c**,**d**), ThT and Dap (**e**,**f**), ThT and MB (**g**,**h**), CR and ANS (**i**,**j**), CR and Dap (**k**,**l**), CR and MB (**m**,**n**), ANS and Dap (**o**,**p**), ANS and MB (**q**,**r**), Dap and MB (**s**,**t**). Inserts show the difference in bound dye concentration when they are both mixed with fibrils as opposed to being mixed separately. The difference is displayed as a percentage of total dye present in the initial sample.

Examining single dye and dye-pair binding to insulin fibrils reveals that in every single case, there was an interference between these amyloidophilic molecules, even when the dye-dye interactions were accounted for. For ThT, the presence of every other dye increased its binding to fibrils (Fig. 2a–h), with the most notable change seen in the ThT-CR pair, where almost all ThT is bound. For CR (Fig. 2a,b,i–n), there were only very minimal changes in bound concentration and the vast majority of it remained on the fibril’s surface, as it has a high binding affinity^37^. For ANS (Fig. 2c,d,i,j,o-r), there were small changes when paired with ThT or MB, but a noticeable decrease with CR and Dap. For Dap (Fig. 2e,f,k,l,o,p,s,t), there was an increase in bound concentration with ThT and MB, and a decrease with CR and ANS. For MB (Fig. 2g,h,m,n,q–t), there were small increases with ThT, ANS and Dap, and a larger one with CR. The compensation curves nearly perfectly mimicked the absorbance difference curves, except in the cases of ThT-MB, ANS-MB and Dap-MB.

**Figure 3:**
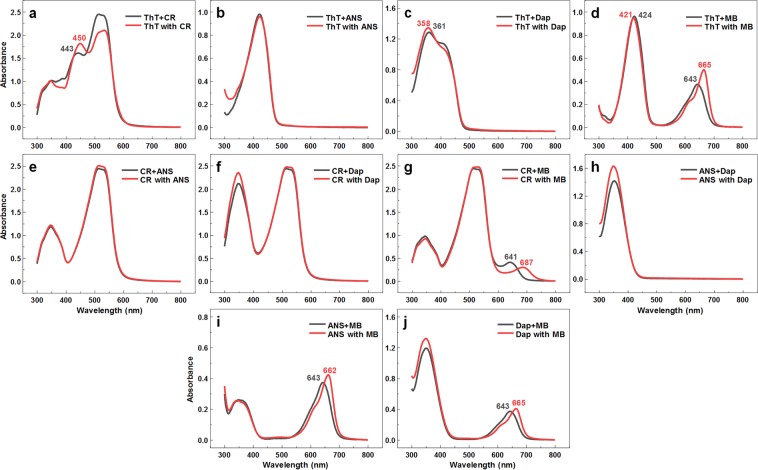
Absorbance spectra of dyes mixed with insulin fibrils. Comparison between absorbance spectra when the dyes were mixed with fibrils separately and then the spectra were combined (marked as Dye + Dye) and when both dyes were mixed with fibrils (marked as Dye with Dye) for ThT-CR (**a**), ThT-ANS (**b**), ThT-Dap (**c**), ThT-MB (**d**), CR-ANS (**e**), CR-Dap (**f**), CR-MB (**g**), ANS-Dap (**h**), ANS-MB (**i**) and Dap-MB (**j**) dye pairs. Color-coded numbers indicate the absorbance peak wavelengths (nm), where there is a shift in the peak’s position.

The absorbance spectra of dyes when they are bound to fibrils was also examined (Fig. 3). When comparing the combined spectra of single dyes bound to fibrils with the spectra when both dyes are present, we see that in some of the cases, namely ThT-ANS (Fig. 3b), CR-ANS (Fig. 3e), CR-Dap (Fig. 3f) and ANS-Dap (Fig. 3h), there is virtually no difference between their peak wavelengths and only a small change in the case of ThT-Dap (Fig. 3c). However, there are very clear differences when comparing ThT-CR (Fig. 3a), ThT-MB (Fig. 3d), CR-MB (Fig. 3g), ANS-MB (Fig. 3i) and Dap-MB (Fig. 3j). In the ThT-CR pair, the ThT-related peak position shifts from 443 nm to 450 nm and in the ThT-MB pair - from 421 nm to 424 nm. In every case where MB is present, the MB-specific peaks experience a drastic change: from 643 nm to 665 nm in the ThT-MB pair, from 641 nm to 687 nm in the CR-MB pair, from 643 nm to 662 nm in the ANS-MB pair and from 643 nm to 665 in the Dap-MB pair.

### Dye fluorescence

**Figure 4:**
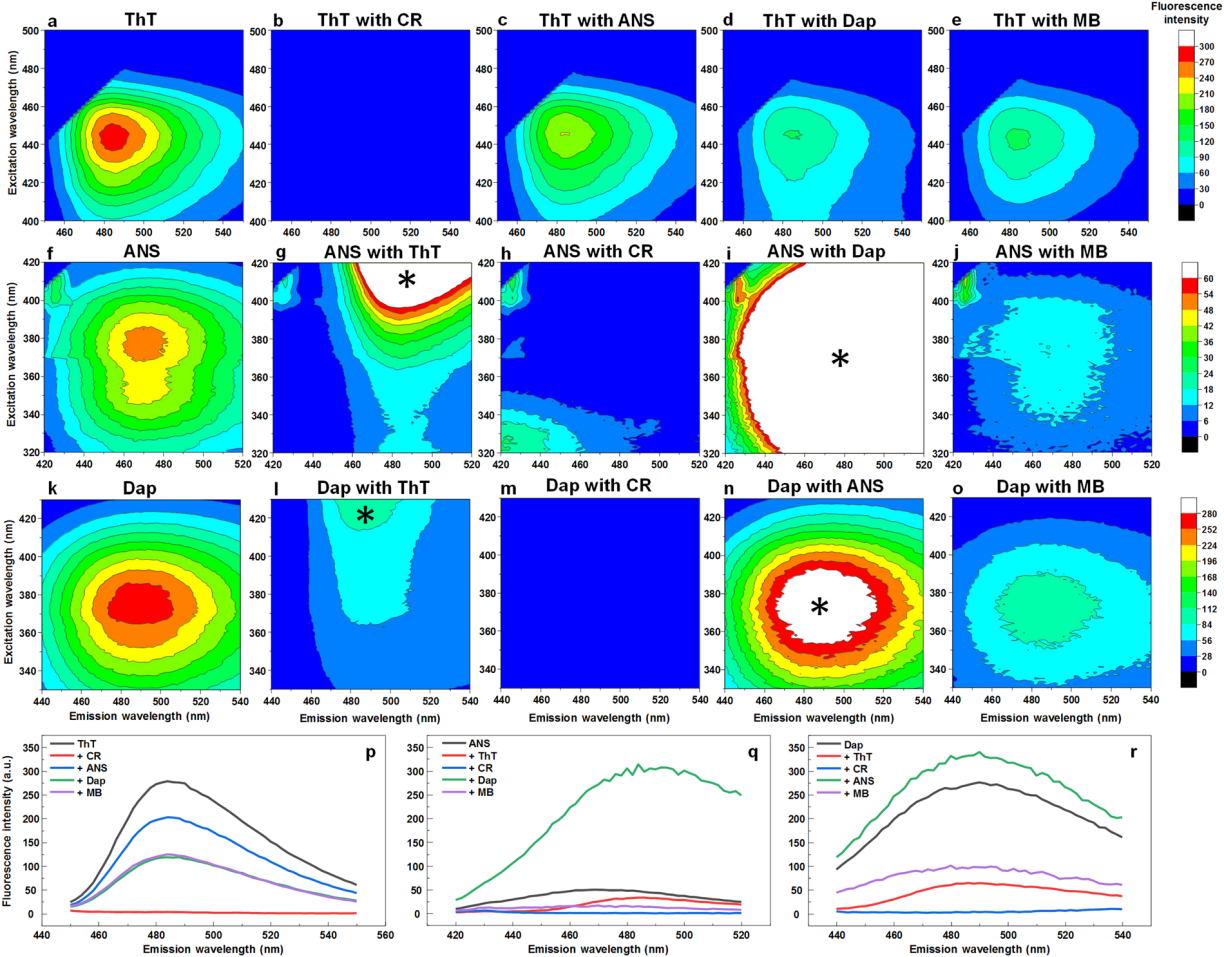
Excitation-emission matrices of ThT, ANS and Dap interacting with CR, MB and each other on insulin fibrils. EEM centered on the ThT emission signal maxima of single ThT (**a**) and in pairs with CR (**b**), ANS (**c**), Dap (**d**) and MB (**e**). EEM centered on the ANS emission signal maxima of single ANS (**f**) and in pairs with ThT (**g**), CR (**h**), Dap (**i**) and MB (**j**). EEM centered on the Dap emission signal maxima of single Dap (k) and in pairs with ThT (**l**), CR (**m**), ANS (**n**) and MB (**o**). Single excitation wavelength emission spectra of ThT at 440 nm (**p**), ANS at 380 nm (**q**) and Dap at 375 nm (**r**) mixed with other dyes. The * symbol represents fluorescence emission signals resulting from another dye in the solution.

The excitation-emission matrices of ThT (Fig. 4a–e), ANS (Fig. 4f–j) and Dap (Fig. 4k–o), as well as single wavelength fluorescence spectra (Fig. 4p–r) with fibrils separately and in pairs were acquired and corrected for the primary and secondary inner filter effect in order to examine any possible effects of dye interference on their fluorescence intensity. In the case of ThT, when comparing the EEM of the dye alone with fibrils (Fig. 4a) and mixed with other amyloidophilic molecules, we observe an almost complete fluorescence quenching in the ThT-CR pair (Fig. 4b). There is also a substantial quenching effect seen with all other three tested molecules (Fig. 4c–e). In the case of ANS (Fig. 4f), CR completely quenches its fluorescence (Fig. 4h) and MB partially reduces its emission intensity (Fig. 4j). ANS-ThT (Fig. 4g) and ANS-Dap (Fig. 4i) pairs are more difficult to analyze, as their EEM spectra partially overlap, however, in the ANS-ThT pair we see the ANS EEM maxima position intensity is considerably reduced. The ANS-Dap pair has a similar intensity when compared against a sum of separate ANS and Dap spectra (327 and 334 a.u. respectively at the most intense EEM position). For Dap (Fig. 4k), the addition of ThT, CR or MB greatly quench its fluorescence (Fig. 4l,m,o) and there is virtually no effect when combined with ANS (Fig. 4n), as mentioned previously. There is no observable fluorescence emission intensity maxima shift in any of the cases, where these are no overlapping spectra from other dye molecules.

### ThT and CR interaction

**Figure 5:**
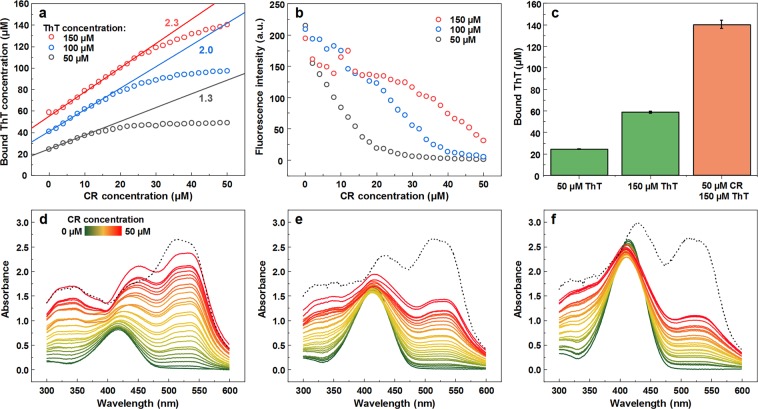
Titration of ThT-insulin fibril solutions by CR. Bound ThT concentration dependence on the concentration of CR present in solution (**a**), where color-coded numbers indicate slope values. ThT fluorescence intensity dependence on the concentration of CR present in solution (**b**). Bound ThT concentration when fibrils are mixed with different dye concentrations (**c**). Absorbance spectra of ThT-CR-insulin fibril solutions when 50 (**d**), 100 (**e**) and 150 (**f**) µM of ThT is present. The black dotted line represents the calculated sum absorbance spectra of separately bound ThT and CR. The absorbance spectra are corrected by subtracting a spectrum of insulin fibrils. Each data point is the average of three measurements.

Seeing as the ThT-CR pair results in both the strongest quenching effect, as well as the most extreme change in ThT binding affinity, these dyes were subjected to further examinations. Fibril samples containing 50, 100 and 150 µM final ThT concentrations were titrated with CR. When the sample contains 50 µM ThT, rising the concentration of CR initially results in a linear increase in the concentration of bound ThT (Fig. 5a), followed by a gradual shift to a plateau, when almost all ThT molecules become bound. The slope of the linear part indicates that one CR molecule causes, on average, an additional binding of 1.3 ThT molecules. When the same titration experiment is conducted with 100 µM ThT (Fig. 5a), the linear section is longer and the slope value is 2.0, with all ThT eventually becoming bound. Finally, in the case of 150 µM ThT (Fig. 5a), the linear part is even further extended, with a slope value of 2.3. Now, however, 50 µM of CR does not cause all of ThT to become bound and it seems that more CR molecules would be needed for this to occur, as a plateau is not reached.

Examining how the gradual increase in CR concentration affects the fluorescence intensity of ThT (Fig. 5b) reveals that when the concentration of ThT is low, adding CR very quickly causes a massive drop in fluorescence emission intensity. When there is 100 µM ThT present, the fluorescence intensity dependence on CR concentration gains a sigmoidal shape and the intensity does not experience a significant drop at low CR concentrations. In the case of 150 µM ThT, there is an even longer initial plateau and the fluorescence intensity does not become completely quenched at the highest CR concentration. The total bound ThT at the highest ThT-CR concentrations tested is significantly higher than the amount of bound ThT when in the absence of CR (Fig. 5c).

Measuring the absorbance spectra of all samples used in the titration experiment reveals that in the case of 50 µM ThT (Fig. 5d), the absorbance maxima at 417 nm (when only free and bound ThT is present), begins to shift towards 450 nm after all ThT becomes bound. Such a shift is not evident under the two larger ThT concentrations (Fig. 5e,f), where the dye does not become fully bound at lower CR concentrations. This peak shift could be caused by an increasing amount of normally bound CR, which is not associated with ThT, as the ThT-CR interactions become saturated. If we calculate what the spectrum of bound ThT and CR was if they were bound separately with no interaction with one another and compare it to the highest ThT-CR concentration spectra, we observe an increasing disparity between them as the concentration of ThT rises. The most notable differences are observed for peaks in the 500–550 nm range associated with CR bound to insulin fibrils.

## Discussion

The examination of dye molecule interactions reveals that no interference is seen in only a few select cases. In other cases, the combined solution absorbance spectra are lower or different than of summed separate dye solution spectra, suggesting the possibility that these molecules interact with one another in solution. In the case of ThT-CR and CR-MB we even see a shift in absorbance maxima positions, which may be due to dye-complex formation. This CR interaction with ThT and MB is interesting, as CR does not seem to interact with other dye molecules, such as ANS or Dap. Another interesting case is the considerable interference of MB, which is a potential amyloidosis inhibitor^35^, with CR, ANS and Dap and to a lesser extent with ThT. These interactions have to be taken into account when examining amyloid fibril formation with more than one dye or when a potential inhibitor molecule possesses a similar scaffold to one that interferes with the dye molecule. Otherwise, a change in the optical properties of a sample could be falsely attributed to changes in fibril concentration.

When examining dye pair binding to fibrils, we observe a quite unusual result. Normally, it would be expected that if two amyloidophilic molecules bind to different parts of the fibril, then there would be no change in bound concentration when they are combined. If the molecules had the same or similar binding position, then there would be a reduction in each of their bound concentration, depending on each molecule’s binding affinity. The strange thing here is that in 7 out of 10 cases, there is an increase in bound dye concentration, which means that the molecules actually aid one another in binding to the fibril’s surface. This could potentially be achieved by either dye-dye interaction or complex formation, as seen in Fig. 1 or by fibril surface changes induced by the binding of dye molecules, such as subtle structural or charge alterations. The absorbance spectra of dyes bound to fibrils indicate that all pairs with MB and the ThT-CR pair experience significant changes. This supports the idea that there is some direct interaction between these molecules when they are located on the surface of fibrils. Examining the fluorescence of ThT, ANS and Dap, we see that in every single pair that contains ThT, its fluorescence is quenched quite dramatically, even though from dye-fibril interactions we know that there is more ThT bound in every case. These results are in line with previously reported observations of a quenching effect for the ThT-CR pair^24^, however, it appears that this effect is not due to less ThT molecules binding, which would be the obvious first conclusion, but due to the existence of another amyloidophilic molecule. The same is true for both ANS and Dap, where the existence of other dye molecules quench their fluorescence emission intensity, except for when the two are paired together. This could be caused by either dye-dye interactions and weak complex formation or their interference while bound nearby on the surface of fibrils, possibly due to energy transfer between each other. There has been a report indicating that Forster-resonance energy transfer (FRET) is possible on amyloid fibrils even if the molecules are not conjugated^38^. Such an event would require the dye molecules to be in close proximity to one another (distances of less than 10 nm), as well as have an overlap between one molecule’s excitation and another’s emission spectra. CR absorbance overlaps with ThT, ANS and Dap emission spectra and the relatively high concentration of bound dye molecules at specific parts of the fibrils could bring them into close proximity. Now while this explains the quenching effect of CR on all three fluorescent dyes, as well as ThT’s effect on ANS and Dap fluorescence and the non-existent quenching between ANS and Dap, this does not explain why ANS, Dap or MB quench the fluorescence of ThT. The emission spectrum of ThT does not match with the absorbance spectra of these three molecules, yet the fluorescence intensity is still considerably reduced. This unlikeliness of a FRET event leads to a conclusion that there has to be some interaction between these molecules, which induces a quenching effect without such energy transfer, which is likely due to a weak-complex formation which changes the dye’s quantum yield.

A further examination of the interaction between ThT and CR on the surface of amyloid fibrils revealed that CR is extremely effective at increasing ThT’s binding affinity. If this was the result of changes to the fibril’s surface, which facilitated more positions for ThT binding, there would be minimal differences between the calculated sum absorbance spectra of bound ThT and CR when compared to the real spectra, which is not the case in either of the tested conditions. This leads to the hypothesis that ThT and CR interact quite effectively on the surface of fibrils, as their complex formation was already reported^26^. However, this complex formation is a lot more complicated, as our results show that one CR molecule can bind more than one ThT molecule. The CR-specific absorbance spectra also change quite drastically when there is a large concentration of available ThT, which suggests that the ThT-CR interaction may be quite strong when bound in close proximity.

One peculiar thing observed during these experiments is the sigmoidal shape of ThT fluorescence intensity dependence on the concentration of CR present. This could be explained by the fact that ThT molecules themselves are capable of self-quenching^28^. Here we can see that there is almost no difference between the fluorescence intensity between ~24 and ~60 µM of bound ThT, when accounted for the inner filter effect. The more ThT is bound, the higher the possibility of fluorescence exists, while at the same time the self-quenching effect becomes stronger and vice versa. This means that if CR forms a complex with some of the bound ThT molecules, the residual bound ThT would experience a smaller self-quenching effect, which would compensate for the decrease in bound dye. The fluorescence intensity begins to decrease only when the self-quenching effect becomes so little that its reduction does not compensate for the loss of normally bound ThT. Another interesting thing to note is that when such a large quantity of dye molecules binds to fibrils, the aggregates change their self-association properties. At higher CR concentrations, an even larger amount of ThT binds to fibrils and it becomes almost impossible to fully separate the dye-loaded aggregates from solution by centrifugation, as they quickly dissociate away from the pellet into solution. Due to this reason, all experiments were limited to a maximum CR concentration of 50 µM.

These results indicate that if a potential new aggregate formation inhibitor or fibril destabilizing drug was tested using amyloidophilic dye fluorescence spectra as a means to track their formation or dissociation, it could very easily lead to false conclusions that the molecule was effective. This is especially true if the molecule in question has a similar scaffold or spectral properties to the tested dyes or if it binds to the surface of amyloid fibrils.

## Conclusions

In a majority of cases, amyloidophilic molecules, such as ThT, CR, ANS, Dap and MB appear to be able to aid each other in binding to the surface of insulin amyloid fibrils and only certain pairings interfere with one another. However, this additional binding does not result in an increased fluorescence of ThT, ANS or Dap. In fact, in most dye pairs, there is a sizeable fluorescence quenching effect, which could be attributed to the interaction of these molecules at the fibril’s surface. Such an effect could lead to false identifications of anti-amyloid drugs if they possess a similar scaffold, spectral properties or binding propensity as the molecules used in this work.

## Supplementary information

Supplementary information.

## Data Availability

The datasets generated during and/or analysed during the current study are available from the corresponding author on reasonable request.
