# Supplementary material for: Amyloidophilic Molecule Interactions on the Surface of Insulin Fibrils: Cooperative Binding and Fluorescence Quenching
Source: Sci Rep. 2019 Dec 30;9:20303. doi: 10.1038/s41598-019-56788-y (PMC6937241; doi:10.1038/s41598-019-56788-y)
Supplement: Supplementary file 1 — Supplementary information. [file 41598_2019_56788_MOESM1_ESM.pdf]

Amyloidophilic Molecule Interactions on the Surface of Insulin Fibrils: Cooperative Binding and Fluorescence Quenching

Mantas Ziaunys, Kamile Mikalauskaite, Vytautas Smirnovas*

Institute of Biotechnology, Life Sciences Center, Vilnius University, Vilnius, Lithuania

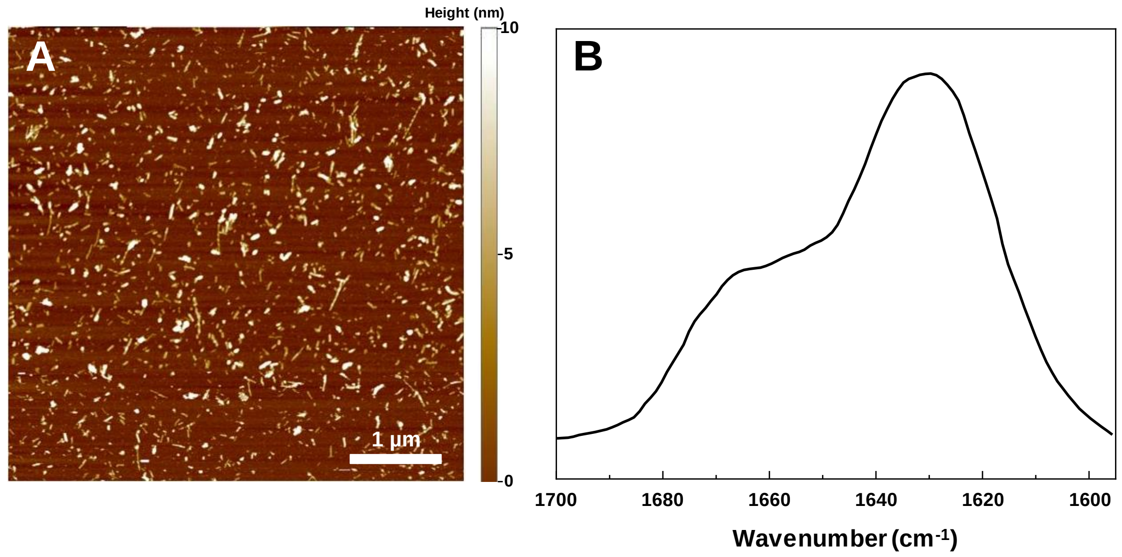

Supplementary Figure S1. AFM image (A) and FTIR spectrum (B) of insulin fibrils prepared as described in the method section. For AFM 20 μL of each sample was deposited on freshly cleaved mica and incubated for 1 minute. The samples were then rinsed with 1 mL of MilliQ water and dried under gentle airflow. AFM images were acquired using Dimension Icon (Bruker) atomic force microscope operating in tapping mode and equipped with a silicon cantilever RTESPA-300 (Bruker). All data processing was performed using Nanoscope software. For FTIR fibrils were separated from buffer solution by centrifugation at 20 000 x g for 30 min and resuspended in 1 mL of D_2O, the procedure was repeated three times. The spectra were recorded using Bruker Alpha spectrometer equipped with a deuterium triglycine sulfate (DTGS) detector. For all measurements, CaF2 transmission windows and 0.1 mm Teflon spacers were used. Spectra were recorded at room temperature. For each spectrum, 256 interferograms of 2 cm^{-1} resolution were co-added. A corresponding buffer spectrum was subtracted from the sample spectrum. All data processing was performed using GRAMS software.
